# SUNCT/SUNA in Pediatric Age: A Review of Pathophysiology and Therapeutic Options

**DOI:** 10.3390/brainsci11091252

**Published:** 2021-09-21

**Authors:** Carlo Alberto Cesaroni, Jacopo Pruccoli, Luca Bergonzini, Giuseppe Quatrosi, Luigi Vetri, Michele Roccella, Antonia Parmeggiani

**Affiliations:** 1 U.O. di Neuropsichiatria dell’Età Pediatrica, Centro Regionale per i Disturbi della Nutrizione e dell’Alimentazione in Età Evolutiva, IRCCS Istituto delle Scienze Neurologiche di Bologna, 40138 Bologna, Italy; carlo.cesaroni@studio.unibo.it (C.A.C.); jacopo.pruccoli@studio.unibo.it (J.P.); luca.bergonzini3@studio.unibo.it (L.B.); 2 Dipartimento di Scienze Mediche e Chirurgiche (DIMEC), Università di Bologna, 40138 Bologna, Italy; 3 Department of Health Promotion, Mother and Child Care, Internal Medicine and Medical Specialties (ProMISE), University of Palermo, 90127 Palermo, Italy; peppe.quatrosi@gmail.com (G.Q.); luigi.vetri@gmail.com (L.V.); 4 Department of Psychology, Educational Science and Human Movement, University of Palermo, 90128 Palermo, Italy; michele.roccella@unipa.it

**Keywords:** SUNCT, SUNA, trigeminal autonomic cephalalgia, autonomic symptoms, children, treatment, ICHD-3, headache

## Abstract

The International Classification of Headache Disorders, 3rd edition (ICHD3) defines Short-lasting Unilateral Neuralgiform Headache Attacks (SUNHA) as attacks of moderate or severe, strictly unilateral head pain lasting from seconds to minutes, occurring at least once a day and usually associated with prominent lacrimation and redness of the ipsilateral eye. Two subtypes of SUNHA are identified: Short-lasting Unilateral Neuralgiform headache attacks with Conjunctival injection and Tearing (SUNCT) and Short-lasting Unilateral Neuralgiform headache attacks with cranial Autonomic symptoms (SUNA). These pathologies are infrequent in children and difficult to diagnose. The authors reviewed the existing literature on SUNCT and SUNA, especially in the developmental age, which describes the pathophysiology in detail and focuses on the therapeutic options available to date. SUNHA-type headaches must be considered on the one hand, for the possibility of the onset of forms secondary to underlying pathologies even of a neoplastic nature, and on the other hand, for the negative impact they can have on an individual’s quality of life, particularly in young patients. Until now, published cases suggest that no chronic variants occur in childhood and adolescents. In light of this evidence, the authors offer a review that may serve as a source to be drawn upon in the implementation of suitable treatments in children and adolescents suffering from these headaches, focusing on therapies that are non-invasive and as risk-free as possible for pediatric patients.

## 1. Introduction

According to the third edition of the International Classification of Headache Disorders (ICHD-3), Short-lasting Unilateral Neuralgiform headache attacks with Conjunctival injection and Tearing (SUNCT) and Short-lasting Unilateral Neuralgiform headache attacks with cranial Autonomic symptoms (SUNA), collectively known as Short-lasting Unilateral Neuralgiform Headache Attacks (SUNHA), are rare primary headache syndromes, which belong to the group of Trigeminal Autonomic Cephalalgias (TACs) [1]. The ICHD-3 defines SUNHA headaches as “attacks of moderate or severe, strictly unilateral head pain lasting from seconds to minutes, occurring at least once a day and usually associated with prominent lacrimation and redness of the ipsilateral eye” [1]. The difference between SUNCT and SUNA lies in the local autonomic signs accompanying the attacks: both ipsilateral conjunctival injection and tearing characterize SUNCT, while either one or the other, along with other cranial autonomic signs, appears in SUNA [1,2,3,4,5,6,7,8,9]. SUNHA, and TACs in general, are relatively rare in adults, and even rarer in the pediatric population [2,3,9,10]; hence, they are poorly recognized. The lack of awareness about this condition among clinicians could expose patients to the consequences of “diagnostic odysseys” [10]. Indeed, a recent paper by Groenke et al. [11] highlighted that undiagnosed SUNHA may lead patients to undergo unnecessary procedures, such as misguided dental treatment, with no pain resolution and exposure to lengthy and costly treatments as well as the persistent risk of adverse events. The aim of the present review is to gather evidence from the literature on the significant features of and therapeutic options for SUNHA at the developmental age in order to provide useful clinical information, given the possible impact of a missed diagnosis upon the quality of life of affected patients.

## 2. Materials and Methods

**Figure 1:**
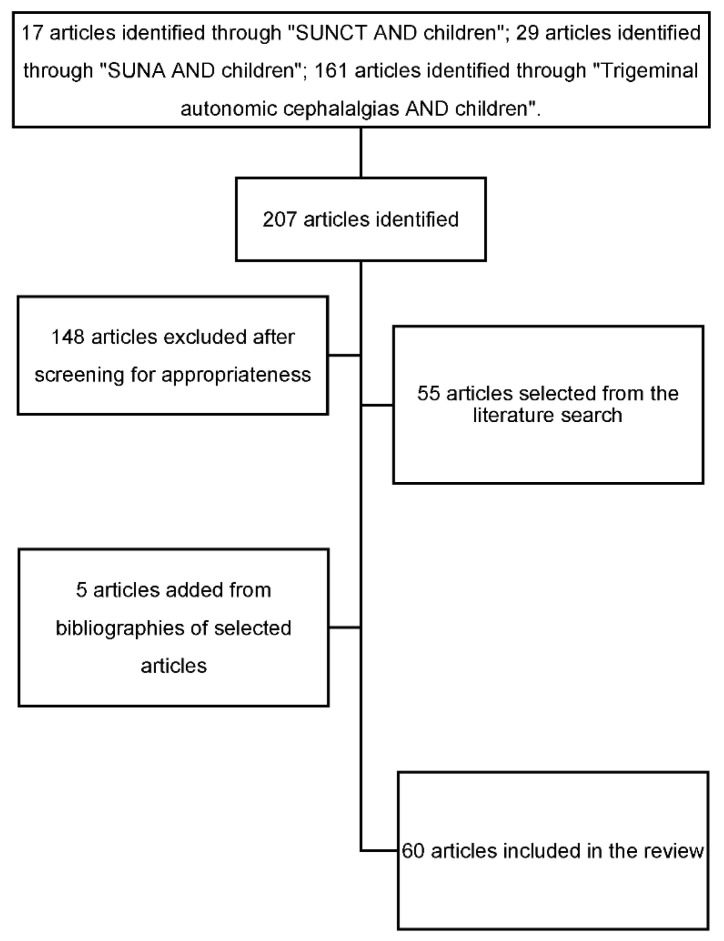
Flowchart of the study.

An extensive web-based search was conducted using PubMed, Web of Science, and Google Scholar. Key search terms included “SUNCT” or “SUNA” or “Trigeminal autonomic cephalalgias”, and “children”, and variations, combined with study filters for original research, case reports and case series. A total of 207 articles were identified. All articles were screened manually for content appropriateness. Additional articles were identified from the reference lists of screened papers. Duplicates were removed. At the end of the selection process, a total of 60 articles were included in this review, including 7 case reports on 19 patients suffering from pediatric SUNHA. Papers published in English (59) and Spanish (1) were reviewed. The flow chart of the study is shown in Figure 1.

## 3. Results

### 3.1. Etiology

Both the idiopathic and secondary form of SUNHA have been described in children. Among the nineteen cases of pediatric SUNHA reported in the literature, thirteen patients had normal brain and orbit MRI, whereas the remaining six had abnormal imaging findings, though not clearly related to TACs (Table 1). Evidence in the literature suggests that SUNHA are primary idiopathic headache syndromes [1,12,13]. However, SUNCT-like syndromes caused by intracranial lesions are reported among adults [3,12], as shown in Table 2. These encompass (1) posterior fossa pathologies, such as pilocytic astrocytoma, cavernous hemangioma, arteriovenous malformation, dorsal–lateral brainstem ischemic lesions, skull malformation or HIV-related lesions [7,9,13,14,15,16,17,18]; (2) pituitary pathology, such as microadenomas or macroadenomas, with prolactinomas being the most common type [3,4,7,9,12,13,17,19]; (3) neoplastic lesions within the cavernous sinus, venous sinus or the orbits [3,9]; and (4) vascular loops and trigeminal neurovascular conflict, ipsilaterally to the side of the pain [4,9,13].

However, some authors argue that it is challenging to establish a well-defined causal relationship between the headache and the identified lesions, suggesting the possibility that many of them are incidental findings [12,15,20]. Regardless, performing a full neuroradiological investigation with brain MRI is widely recommended in all cases of TACs/SUNHA, even in patients who exhibit typical clinical features and no neurological deficits, since the clinical phenotype of secondary TACs is indistinguishable from the primary forms [4,7,10,15,18,19,20,21]. In particular, detailed imaging of the posterior fossa and pituitary is of paramount importance [7].

**Table 1:** Reports of pediatric SUNCT cases.

Reference	Sex	Age at Onset	Diagnosis	Symptoms	Imaging	Therapy and Outcome
D’andrea, G. & Granella, F. 2001 [2]	F	10 yr	SUNCT	Moderate/severe, right-sided (seldom left-sided), stabbing pain attacks, lasting 2–180 s, 10–180 s per hour over the first 2 months; ipsilateral conjunctival injection, lacrimation and occasional nasal obstruction	Normal MRI and CT	Indomethacin (100 mg daily), other NSAIDs (aspirin, nimesulide, ketoprofen) with no effect; spontaneous remission over 6 months
Blattler, T., Capone Mori, A., Boltshause, E. & Bassetti, C. (2003) [14]	F	11 yr	SUNCT	Moderate/severe, strictly right-sided, sharp pain attacks, lasting 30–60 s, 20 per day; ipsilateral conjunctival injection, lacrimation and salivation	Pylocitic astrocytoma	Indomethacin (100 mg daily): frequency dropped from 20 to 10 per day, with no effect on pain intensity
Sékhara, T., Pelc, K., Mewasingh, L. D., Boucquey, D. & Dan, B. (2005) [16]	M	5 yr	SUNCT	Mostly left-sided, burning or stabbing pain, lasting 2–50 s, 4–6 per hour every 2–3 days; conjunctival injection, lacrimation, nasal congestion	Normal MRI	No medication was administered; spontaneous remission over five months
Ünalp, A. & Öztürk, A. (2008) [18]	M	6 yr	SUNCT	Shooting pain, lasting 5–10 min, 3–4 per day; swelling, rash, ptosis	Normal MRI	Lamotrigine, 25 up to 100 mg/day, with benefit
Sciruicchio, V. et al. (2010) [3]	F	2 yr	SUNCT	Severe, right-sided pain attacks, lasting 5–30 s, 10 per hour, occurring at awakening; impressive ipsilateral conjunctival injection and tearing	Normal MRI	The spontaneous remission within a few hours made prophylactic therapy unnecessary
Zhang, Y et al. (2016) [9]	M	12 yr	SUNCT	Severe, left-sided, lasting 60 s; ipsilateral conjunctival injection and tearing, facial flushing and running nose	Normal MRI	Oral carbamazepine (200 mg daily) discontinued due to an allergic reaction; gabapentin (100 mg) three times daily, pregabalin (75 mg) twice daily, indomethacin (25 mg) three times daily, flunarizine (5 mg) at night, ibuprofen (300 mg) four times daily, topiramate (25 mg) twice daily, methylprednisolone (80 mg) daily, 7–10 L/min of pure oxygen for 10–20 min per day, 2% lidocaine (2 mL) nasal drops, with no changes in the severity or frequency of pain attacks
Qaiser, S., Hershey, A.D., Kacperski, J. (2020) [22]	6 M 7 F	3–18 yr	SUNCT SUNA	13 pts: unilateral, stabbing pain attacks, lasting 1 s–10 m, for more than 3 months; 4 pts: conjunctival injection, tearing 2 pts: tearing, eyelid edema 2 pts: facial swelling, tearing 2 pts: eyelid edema, tearing, facial swelling 1 pt: facial swelling 1 pt: eyelid edema, injection, tearing 1 pt: facial flush, tearing	8 pts: normal MRI 1 pt: left cerebellar hemangioma 1 pt: multifocal demyelinating lesions 1 pt: Chiari I post surgical decompresson 1 pt: cavum septum pellucidum post fenestration 1 pt: low lying cerebellar tonsils	7 pts: indomethacin (1 mg/kg with max 150 mg/day), 5 cases had resolution of attacks 2 pts: oxygen, good response 1 pt: cyprohepatine, non resp 1 pt: amitriptyline, non resp 1 pt: topiramate, good response 1 pt: lost to follow-up

Abbreviations: pts = patients, pt = patient.

**Table 2:** Lesions associated with SUNCT-like syndromes.

Posterior Fossa Pathologies	Pituitary Pathology	Cavernous Sinus/Orbits	Other
Pilocytic astrocytoma Cavernous hemangioma Arteriovenous malformation Basilar impression Dorsal–lateral brainstem ischemic lesions Skull malformation HIV-related lesions Congenital skull bone malformations (e.g., osteogenesis imperfecta) Ischemic infarction Cysts Vascular malformations or venous angioma of the cerebellopontine junction Brainstem angiocavernoma	Micro/macroadenomas (prolactinomas the most frequent)	Neurofibromatosis type 2 Intracranial intraorbital metastasis Extracranial intraorbital cystic tumors Invasion of the cavernous sinus by macroprolactinomas	Vascular loops and trigeminal neurovascular conflict Eye trauma Leiomyosarcoma of the venous sinus

### 3.2. Clinical Features

Pediatric onset SUNHA headaches have been reported in 19 patients (Table 1). Among them, the age at onset varied greatly, ranging from 2 to 18 years of age (mean and median age at onset: 9.9 and 11 years of age, respectively). With regard to the adult population, some authors suggest a higher prevalence of SUNCT in males (with a gender ratio of 17:2) and of SUNA in females [6,7,14,18]. Similar evidence cannot be gathered from the pediatric series, since an equal male and female distribution is described for each headache. Even though it is generally acknowledged that headache features may differ between adults and children, due to different stages of brain development and myelination, all the pediatric cases of SUNHA described in the literature report attacks resembling the ones described among adults. However, the limited number of cases reported so far may not exhaustively express the clinical variability of the disease [2,4,23].

The main clinical features of SUNHA are: (1) short-lasting, very frequent attacks of unilateral stabbing pain, which start and cease abruptly; (2) rapidly developing ipsilateral autonomic signs, due to cranial parasympathetic activation (lacrimation, rhinorrhea, nasal congestion and eyelid oedema) and sympathetic hypofunction (ptosis and miosis) [1,2,4,13,14,15,17]. SUNCT can only be diagnosed when both conjunctival injection and tearing occur; otherwise, the cardinal clinical features of SUNA and SUNCT are similar [1,17]. The attack frequency during the symptomatic phase varies greatly among, or even within patients: they may be as infrequent as once a day, or less, up to more than 60 per hour [4].

According to the ICHD-3, SUNCT and SUNA can be further subdivided into episodic or chronic forms, which are characterized by attacks occurring for more than 1 year without remission or with remissions lasting less than three months [7] (Table 3). Episodic SUNCTs have been reported in the pediatric population [3,9,16]; conversely, chronic forms have not been described so far, although specific long-term follow-up data were not always available in the case reports considered in the present review.

In recent years, the issue of whether SUNCT and SUNA are distinct entities or not has been debated: some authors hypothesize that the former may be a subset of the latter [1,6,7,12,17]; other authors suggest, instead, that differentiating between SUNCT and SUNA does not appear to be clinically relevant [11].

#### 3.2.1. Pain

SUNA and SUNCT are usually considered side-locked headaches, even though a spreading of the pain to the contralateral side has been described in some cases [2,4]. The pain is usually maximal in the ophthalmic distribution of the trigeminal nerve, involving the nose, eyes, or head around the area of the scalp, but can radiate to any part of the head; it has an excruciating intensity and a neuralgic quality, being sharp and stabbing [4,5,13,24].

In the case of infants, toddlers, or children who are unable to self-report symptoms, some information may be gathered differently. Pain localization and intensity may be inferred from such behavioral signs as the association of crying and beating of the head [3], or even by asking patients to describe their headache with drawings or figures. The repetition of drawings over time may also be useful to better understand the clinical course of symptoms [25].

**Figure 2:**
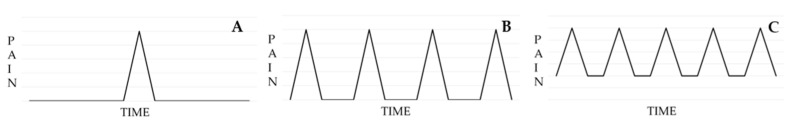
Attack forms in SUNHA headache: single stabs (**A**), groups of stabs (**B**), saw-tooth pattern (**C**). Abbreviations: SUNHA (Short-lasting Unilateral Neuralgiform Headache Attacks).

Attacks may take any one of three forms: (1) single stabs, which are usually short-lived, (2) groups of stabs, and (3) a saw-tooth pattern, a longer attack comprising many stabs between which the pain does not go away or in which pain persists, but less acutely (Figure 2). Identifying the last type can be a diagnostic challenge and may result in a misdiagnosis, typically with cluster headache. It is also important not to mischaracterize a prolonged group of stabs as a single attack, as this could contribute to confusing the diagnosis [7,19,26]. Triggering by environmental stimulants, as in trigeminal neuralgia, has also been reported for SUNCT syndrome in the adult population: mechanical movements of the neck, cold or hot weather, emotional stress, and instant postural changes may precipitate the headache. The majority of patients can precipitate attacks by touching certain trigger zones belonging to trigeminal innervation and, occasionally, even to extra-trigeminal territories. Precipitants include touching the face or scalp, washing, shaving, eating, chewing, brushing teeth, talking, and coughing [4,18,27,28]. Triggering factors were not identified in any of the pediatric case reports considered in the present review.

Between attacks, most patients are completely pain-free, and only seldom do attacks occur at night, even though poor sleep quality is reported [11,27]. Prodromes have rarely been reported in the literature, with only three adult case reports documenting aura as part of the manifestation of SUNCT attacks [11,29,30].

#### 3.2.2. Autonomic Signs

Conjunctival congestion is the most common autonomic symptom and may be accompanied by lacrimation (SUNCT); forehead and facial sweating, facial flushing, nasal congestion, rhinorrhea, and ptosis may also be observed. Increased intraocular pressure on the symptomatic side and vascular engorgement and swelling of the eyelids (eyelid edema) with decreased palpebral width (pseudoptosis) may occur in the course of headache attacks and are generally located unilaterally in the orbital/periorbital region; conversely, temporal, nasal, mandibular, frontal, palatal and periauricular localizations are less frequent [5,17,18,26]. Ictal changes in pupil diameter are rare in SUNCT [17]. Emerging evidence suggests that in SUNHA, the cranial autonomic symptoms tend to follow the site of the pain, with ocular symptoms occurring predominantly with pain in V1 and nasal symptoms with pain in V2 and V3 [13].

#### 3.2.3. Differential Diagnoses

Recent evidence suggests that SUNHA and other TACs may have higher prevalence in children than is normally perceived and should be included in the differential diagnosis when pediatric patients with headache show strict unilateral autonomic symptoms. Otherwise, these young patients may experience the consequences of several inconclusive, stressful, and expensive diagnostic tests or therapeutic approaches over time. [10,22].

The features of SUNCT and SUNA overlap with other TACs, such as cluster headache or episodic and chronic paroxysmal hemicrania; trigeminal neuralgia and idiopathic stabbing headache (‘jabs and jolts syndrome’) are among the main differential diagnoses as well [18,22]. It has been suggested that criteria to perform a differential diagnosis between these conditions and SUNHA in the pediatric population may be broader than those established for adult patients [22]. Indeed, some cardinal features, such as the duration of attacks and responsiveness to indomethacin, may evolve with the developing brain. [22].

In general, SUNCT and SUNA are phenotypically different from the other TACs in that (1) SUNHA headaches have the shortest attack duration and the highest attack frequency [13,19]; (2) pain in SUNHA headaches most commonly occurs as sharp, stabbing pain in the first division of the trigeminal nerve, which is distinct from the longer-lasting and typically boring pain of paroxysmal hemicrania and cluster headache [7].

Evidence that points toward SUNCT/SUNA acting against trigeminal neuralgia include: the prominent distribution of pain in the ophthalmic division of the trigeminal nerve, prominence of the autonomic features, a longer duration of attacks, and the absence of a refractory period to trigger factors [3,4,6,7,11,19,20]. The differential diagnosis may be particularly challenging in children [31].

In contrast to cluster headache, no beneficial effect of oxygen, sumatriptan or verapamil has been reported, and single attacks are shorter in duration [3,19,20]. In primary stabbing headache, cranial autonomic features are absent, and the site and radiation of pain often varies greatly between attacks; moreover, the majority of the attacks tend to be spontaneous [4,7].

In contrast to paroxysmal hemicrania, in SUNCT/SUNA headaches, attacks are shorter and more frequent, and there is no reproducible indomethacin effect [7,31,32]. The indomethacin responsiveness usually allows a diagnostic distinction; however, it lacks critical biomarkers to address the separation of responders versus non-responders and is clinically and anecdotally driven.

### 3.3. Pathophysiology

The clinical features of SUNCT and SUNA are thought to depend on the activation of the trigeminal system, which causes pain, and the central disinhibition of the trigeminal autonomic reflex, responsible for the autonomic signs. The pathogenic mechanism seems to be linked to an activation of the posterior hypothalamus: on the one hand, this activation determines heightened sensitivity to various noxious stimuli due to the connection to the pain-modulating system via the trigemino-hypothalamic tract; on the other hand, the “internal trigger” area in the posterior hypothalamus, along with the trigeminal nucleus caudalis, stimulates the superior salivatory nucleus, from which parasympathetic efferent fibers travel in the greater superficial petrosal nerve toward the lacrimal system, nasal mucosa and blood vessels, liberating vasoactive intestinal peptide (VIP) and resulting in autonomic signs [4,5,7,11,12,13,16,17,18,20].

This pathophysiological model is consistent with evidence of common clinical features between SUNHA and other TACs. In TACs, posterior hypothalamic activation has been reported (i.e., PH, CH, SUNCT and HC), and with evidence provided by functional imaging studies and deep brain stimulation in the posterior hypothalamic region [9,13,33]. Indeed, functional MRI has identified ipsilateral or bilateral hypothalamic activation in adults with SUNCT [12,16,34,35,36]. However, it can hardly explain other key clinical features of SUNCT/SUNA, including the neuralgiform type of pain, the very short duration and high frequency of the attacks and the absence of a refractory period, which are unique characteristics of these disorders amongst the TACs [13].

Recent findings suggest that peripheral mechanisms, such as trigeminal vascular/tumoral compression or infectious disruption, could be involved in the pathogenesis of the disease through an ephaptic, pain generation mechanism. This evidence is supported by immediate, dramatic pain relief in series of pediatric and adult patients undergoing tumoral debulking and microvascular decompression, respectively, and by BOLD-fMRI studies failing to show any hypothalamic change in secondary cases of SUNCT [5,14,20]. Regardless, the possible persistence of autonomic features suggests that central pathways could remain active in these cases [20].

Ultimately, clinical features occurring in SUNHA may be the result of different degrees of interaction between peripheral and central pathogenic mechanisms [13].

A growing interest in the influence of circadian rhythms in the pathophysiology of SUNCT is developing. Attacks occur predominantly during the daytime, but significant nocturnal clusters have been reported in an adult patient with comorbid, prolactin-secreting microadenoma; in this case, high prolactin (PRL) levels were documented during nocturnal SUNCT episodes [37,38]. A possible neuromodulation for PRL of sensory neurons in the trigeminal ganglia was hypothesized in light of the unlikely compressive etiology and of previous evidence of these receptors in trigeminal ganglia in rats [39]. Thus, the authors suggested that clinicians should evaluate nocturnal PRL levels for all patients suffering from nocturnal SUNCT episodes.

### 3.4. SUNCT/SUNA Therapy

The recognition and distinction of the diverse subcategories of TACs have relevant clinical and therapeutic implications. These syndromes are considered to be some of the most painful ones known to humankind; it is essential to underline the importance of recognizing them and starting selective treatments [4]. Since the acute attacks are very short in time, the needed therapy frequently reveals itself to be of limited help in these conditions. Thus, the management of SUNCT/SUNA in children is mainly based on prophylactic treatment. Very few studies address the treatment of this type of headache in the developmental age; most of them are case reports and case series [2,3,9,14,16,18,22]. There is some limited controlled trial evidence on the use of topiramate in SUNCT [12,27]. We have to consider prophylactic medication for children who report more than four headaches per month.

#### 3.4.1. Lamotrigine

Lamotrigine (LTG) was first synthesized in the early 1980s. Since its market authorization, it has been increasingly used in the treatment of pediatric epilepsy [40]. LTG is a member of the sodium channel blocking class of antiepileptic drugs [41]. It has been proposed that the main features of SUNCT syndrome can be related to an activation of the trigeminal–autonomic reflex. This reflex pathway consists of a brainstem connection between the trigeminal nerve and facial parasympathetic outflow [2,42]. Lamotrigine stabilizes the neuronal sodium channel [43] and may suppress the excessive release of glutamate, which is an NMDA neurotransmitter involved in the antinociceptive pathway responsible for the establishment of chronic pain [2]. In studies regarding animal models, lamotrigine induced dose-dependent analgesia of acute and chronic pain [44] and, immediately after sciatic nerve transection, dramatically reduced the development of neuropathic pain [2]. Lamotrigine has proven effective in the treatment of trigeminal neuralgia and other neuropathic pains [45]. Based on a few reports, pediatric SUNCT could be treated with lamotrigine (25 up to 100 mg/day) [17,18]. Lamotrigine should be initiated at a low dose and titrated slowly with close patient monitoring to recognize the minimum effective dose and avoid serious adverse effects. Long-term treatment with increased doses of lamotrigine may be necessary to control SUNCT syndrome symptoms [46].

#### 3.4.2. Topiramate

Topiramate (TPR) is an antiepileptic drug with positive efficacy and safety for older children and adults with epilepsy [47]. It has been approved for migraine prevention in adults in Europe since 2003 and in the United States since 2004 [48,49]. TPR has been approved for the treatment of children as young as 2 years old for partial-onset seizures and primary generalized tonic–clonic seizures as add-on therapy. TPR is a first-line option for the treatment of migraines in adults. In March 2014, the U.S. Food and Drug Administration (FDA) approved topiramate for migraine prevention in the population aged 12 to 17 [50]. Evidence suggests that TPR may be helpful in the treatment of binge eating disorder [51] in children and adolescents. TPR is the first and only medication currently approved for use in migraine patients of 12 years and older. Topiramate may improve pediatric migraine via a series of direct effects on neurotransmission in brain cells. Through the blockade of sodium and calcium channels, topiramate is able to modulate the abnormal function of these channels in migraines. As a result, neurotransmitter release and blood vessel dilatation appear to decrease [52,53]. A decrease in GABA-mediated inhibition is thought to play a role in migraine development. The modulation of GABA receptors simply results in an enhancement of GABA-mediated inhibition [54]. An increase in glutamate-mediated excitation is also proposed to play a key role in the development of migraines. Therefore, the inhibition of AMPA and kainate currents result in less excitation because trigeminal firing is inhibited [52]. The inhibition of carbonic anhydrase activity results in an overall decrease in the excitatory neurotransmission and an increase in inhibitory neurotransmission [55,56]. According to the existing literature, to minimize the potential side effects, the dose has to be slowly increased, typically by increments of 12.5 mg every 2 weeks until headache responsiveness is obtained, or else side effects develop [57]. Two articles specifically addressed the treatment of pediatric SUNCT with topiramate. The first study [9] treated a 12-year-old patient with severe SUNCT with topiramate at a dosage of 25 mg twice daily, increased up to 150 mg per day, without obtaining a significant improvement of the symptoms. In the second study [22], two additional patients of developmental age (13 and 18 years, respectively) were treated with topiramate. The younger patient responded positively to the treatment; the older patient was lost to follow-up. Together, these studies indicate that the evidence supporting the use of topiramate in the treatment of pediatric SUNCT is still scarce.

#### 3.4.3. Other Treatments

Other therapies are described for possible use in the treatment of this pathology but are not considered effective in a unique way. In particular, a case series [29] underscores the efficacy of indomethacin (1 mg/kg with max 150 mg/day) on almost all the patients tested and shows the use of pure oxygen as decisive for the symptoms. In other case reports, however, oxygen (10–20 min per day) has been proven to be useless for resolving acute attacks [9], while indomethacin (25 mg three times daily) appears to be useful to reduce the frequency of attacks, without affecting the painful symptoms [14] (Table 2). In pediatric age, if indomethacin is started, a response should occur within 72 to 96 h. If no significant change is noted with the maximum dosage of indomethacin after 2 weeks, the diagnosis should be reconsidered and the drug discontinued [58]. Zhang et al. [9] report a case of good response to botulinum toxin treatment, through the administration of a total dose of 70 U around the orbit, temporal area, and upper gum.

## 4. Conclusions

SUNCT/SUNA headaches in pediatric-age patients, although rare and described in few case reports or case series [2,3,9,14,16,18,22], are TACs that should be taken into serious consideration. The anamnestic collection in children is particularly complex and cannot be solely based on the information provided by parents. In fact, it is necessary to gain the trust of the young patient to gather as much information as possible. Identifying the clinical characteristics can be complex, but the right intuition can allow us to identify neoplastic pathologies [38]. Diagnosing a primary form of SUNCT/SUNA allows us to use a targeted therapy to treat the symptoms, while always considering any side effects of the drugs to be used. Symptoms can disappear even without using drug therapy [3,16]. The difficulty in making a correct diagnosis may cause a huge number of neuro-imaging tests, including CT of the brain, especially in the course of an acute attack, or MRI, and the use of numerous therapies that are often ineffective. In either case, the families of affected patients incur enormous expenses, whereas the pathology could be resolved using such targeted drugs as topiramate [9,22] or lamotrigine [48,49]. Since the use of these drugs may be complicated by relevant, despite infrequent side effects, clinicians should carefully apply part of their consultation time to building sound therapeutic relationships, especially with pediatric patients and their parents. This may reduce the risk of dropouts, which is a relevant issue in managing pharmacological treatments in pediatrics. Up to now, for the cases described in the literature and the articles analyzed in this review, we have not identified any cases of SUNHA in pediatric-age patients which gave rise to episodes of chronic disease.

Further progress in non-invasive therapeutic approaches, especially aimed to address the needs of patients of developmental age, and targeted studies on these issues would help clinicians to tackle pathologies that considerably impact the quality of life of patients in the best way.

## Figures and Tables

**Table 3:** Diagnostic criteria for SUNHA (ICHD-3).

**SUNHA** *Diagnostic criteria:* At least 20 attacks fulfilling criteria B–D; Moderate or severe unilateral head pain, with orbital, supraorbital, temporal and/or other trigeminal distribution, lasting for 1–600 s and occurring as single stabs, series of stabs or in a saw-tooth pattern. At least one of the following five cranial autonomic symptoms or signs, ipsilateral to the pain: ○ Conjunctival injection and/or lacrimation; ○ Nasal congestion and/or rhinorrhoea; ○ Eyelid oedema; ○ Forehead and facial sweating; ○ Miosis and/or ptosis. Occurring with a frequency of at least one a day; Not better accounted for by another ICHD-3 diagnosis.	**SUNCT** *Diagnostic criteria:* Attacks fulfilling criteria for SUNHA, and criterion B below; Both of the following, ipsilateral to the pain: ○ Conjunctival injection; ○ Lacrimation (tearing).	**Episodic SUNCT/SUNA** *Diagnostic criteria:* Attacks fulfilling criteria for SUNCT or SUNA and occurring in bouts; At least two bouts lasting from 7 days to 1 year (when untreated) and separated by pain-free remission periods of at least 3 months.
	**SUNA** *Diagnostic criteria:* Attacks fulfilling criteria for SUNHA, and criterion B below; Not more than one of the following ipsilateral to the pain: ○ Conjunctival injection; ○ Lacrimation (tearing).	**Chronic SUNCT/SUNA** *Diagnostic criteria:* Attacks fulfilling criteria for SUNCT or SUNA, and criterion B below; Occurring without a remission period, or with remissions lasting less than three months, for at least one year.

## Data Availability

Not applicable.
